# Changes in Working Patterns in a Community Eating Disorder Service; the Impact of the COVID-19 Pandemic and Beyond

**DOI:** 10.1192/bjo.2025.10508

**Published:** 2025-06-20

**Authors:** Parvathy Mohandas, Maggie McGurgan

**Affiliations:** 1 Northern Ireland Medical and Dental Training Agency, Belfast, United Kingdom; 2 Northern Health and Social Care Trust, Antrim, United Kingdom

## Abstract

**Aims:** One of the aims of this project was to look at changes in working patterns within our local Eating Disorder service over time, paying particular attention to changes in rates of accepted referrals, and rates of inpatient admissions during and after the COVID-19 pandemic. A secondary aim was to look at rates of co-morbidity in the local Eating Disorder Population and compare this to the general population of a similar age group. In order to do this, findings for the general population were extrapolated from the Youth Wellbeing Prevalence Study Northern Ireland (YWPS).

**Methods:** Data on accepted referrals was extracted from a locally used electronic systemic LCID which came into use in 2018 in Northern Trust CAMHS. Additional information regarding admissions and co-morbidities were obtained using another electronic system widely used in Northern Ireland, NIECR. Data included referrals accepted between October 2017 and September 2024, and excluded referrals made to the CAMHS Eating Disorder Service which were rejected. Information regarding admissions to Beechcroft, a regional psychiatric inpatient unit for young people under the age of 18, was obtained from medical administration in the Belfast Trust. Data was generated regarding admissions accepted specifically from our Eating Disorder Service.

**Results:** The admission rate (including admissions to adult medical wards) was 13.84% (35–50% UK wide). Of the 64 young people who were admitted to the paediatric ward 11% were male and 89% were female. The average length of admission to A2 was 21.13 days and the average age at the time of admission was 13.64 years old. The rate of Autism/Autistic traits in those Eating Disorder patients admitted to the paediatric ward was nearly 4.4 times greater than the population from the YWPS. Rates of anxiety were increased by more than 4 times. Rates of low mood/depression were increased by more than 11-fold.

**Conclusion:** The hospital admission rate within our service is significantly lower than the UK-wide admission rate for Eating Disorders. There was a sharp increase in referrals to the CAMHS Eating Disorder Service during the COVID-19 pandemic. Following the end of the COVID-19 lockdowns and easing of restrictions there has been a return to almost pre-pandemic levels in terms of referrals. Paediatric admissions and psychiatric admissions increased significantly during the COVID-19 pandemic. Rates of psychiatric admissions for eating disorders are now much lower than pre-pandemic levels. Rates of paediatric admissions for eating disorders remain elevated.

